# Sagittal Craniosynostosis: Comparing Surgical Techniques Using 3D Photogrammetry

**DOI:** 10.1097/PRS.0000000000010441

**Published:** 2023-03-22

**Authors:** Tareq Abdel-Alim, Melissa Kurniawan, Irene Mathijssen, Marjolein Dremmen, Clemens Dirven, Wiro Niessen, Gennady Roshchupkin, Marie-Lise van Veelen

**Affiliations:** Rotterdam and Delft, the Netherlands; From the Departments of 1 Neurosurgery; 2 Radiology and Nuclear Medicine; 3 Plastic, Reconstructive Surgery, and Hand Surgery; 5 Epidemiology; the 6 Pediatric Brain Center, Erasmus MC, University Medical Center; 4 Faculty of Applied Sciences, Delft University of Technology.

## Abstract

**Background:**

The aim of this study was to compare three surgical interventions for correction of sagittal synostosis—frontobiparietal remodeling (FBR), extended strip craniotomy (ESC), and spring-assisted correction (SAC)—based on three-dimensional (3D) photogrammetry and operation characteristics.

**Methods:**

Patients who were born between 1991 and 2019 and diagnosed with nonsyndromic sagittal synostosis who underwent FBR, ESC, or SAC and had at least one postoperative 3D photogrammetry image taken during one of six follow-up appointments until age 6 were considered for this study. Operative characteristics, postoperative complications, reinterventions, and presence of intracranial hypertension were collected. To assess cranial growth, orthogonal cranial slices and 3D photocephalometric measurements were extracted automatically and evaluated from 3D photogrammetry images.

**Results:**

A total of 322 postoperative 3D images from 218 patients were included. After correcting for age and sex, no significant differences were observed in 3D photocephalometric measurements. Mean cranial shapes suggested that postoperative growth and shape gradually normalized with higher occipitofrontal head circumference and intracranial volume values compared with normal values, regardless of type of surgery. Flattening of the vertex seems to persist after surgical correction. The authors’ cranial 3D mesh processing tool has been made publicly available as a part of this study.

**Conclusions:**

The findings suggest that until age 6, there are no significant differences among the FBR, ESC, and SAC in their ability to correct sagittal synostosis with regard to 3D photocephalometric measurements. Therefore, efforts should be made to ensure early diagnosis so that minimally invasive surgery is a viable treatment option.

**CLINICAL QUESTION/LEVEL OF EVIDENCE:**

Therapeutic, III.

Sagittal synostosis is a congenital condition that involves premature fusion of the sagittal suture. This condition results in an elongated (anteroposterior) and narrow (transverse) shape of the head, also known as scaphocephaly. Frontal bossing or formation of an occipital bullet is frequently present.^1^ Compared with other nonsyndromic single-suture craniosynostoses, sagittal synostosis has the highest prevalence, and is estimated to affect one in every 2000 live births worldwide.^1–3^

Sagittal synostosis can affect the functional and aesthetic development of the child. It causes a higher risk of developing intracranial hypertension (ICH), speech and language problems, intellectual impairment, and psychologic difficulties.^4–8^

Different surgical techniques have been described to correct scaphocephaly.^9,10^ In the Erasmus MC, the preferred surgery changed over time from frontobiparietal remodeling (FBR) at 9 to 12 months of age to extended strip craniectomy (ESC) and minimally invasive spring-assisted correction (SAC) before 6 months of age.^10^ However, there is no consensus on the most effective surgical technique.^11–20^

Objective measurements, such as the cephalic index, occipitofrontal head circumference (OFC), and intracranial volume (ICV), are commonly used to evaluate postoperative results.^21–23^ Obtaining these measurements is a cumbersome and time-consuming task, involving manual measurements and traditional imaging modalities. To minimize radiation exposure and discomfort in young patients during follow-up, aesthetic outcomes of surgical interventions are often assessed subjectively by the clinician and parents.^24^ This is problematic in the pursuit of obtaining an objective consensus regarding the best treatment and timing for patients with craniosynostosis. Three-dimensional (3D) photogrammetry is a noninvasive and radiation-free imaging modality that can serve as a useful instrument in this endeavor.

A 3D photogrammetry setup is used to generate a digital 3D model of the patient’s head. Three-dimensional photogrammetry is rapidly gaining popularity in clinical research and has been shown to be a highly reliable, accurate, and safe instrument for reproducible craniofacial shape analysis in both children and adults.^25^

In this study, we examined patients who had at least one postoperative 3D photogrammetry image taken before age 6. This age limit was chosen to balance the number of patients in the follow-up period from older and younger cohorts. It is also during those first 6 years that the sutures play an essential role in the development and growth of the skull, after which appositional growth takes over.^26^ These images were used to analyze cranial measurements and shapes after one of three types of surgical interventions: ESC, SAC, and FBR (Fig. 1). Measurements obtained from 3D photogrammetry images are referred to as 3D photocephalometrics. Operating characteristics and clinical measures were compared based on operating time, blood loss, complications, and signs of ICH.

**Fig. 1.**
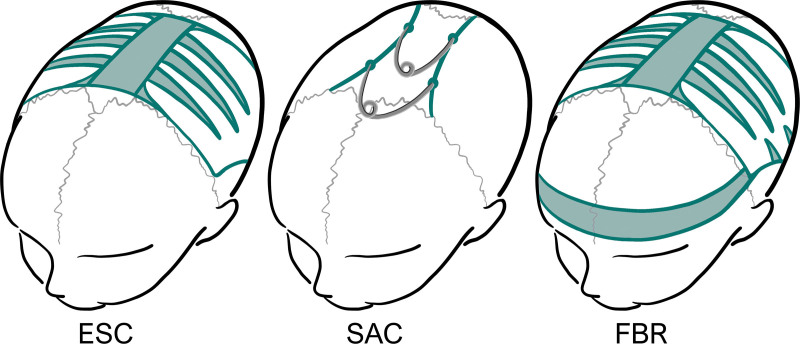
Surgical techniques to correct sagittal synostosis: extended strip craniotomy (*ESC*), spring-assisted correction (*SAC*), and frontobiparietal remodeling (*FBR*).

To stimulate transparent and reproducible research, the framework that was developed and used for mesh visualization, registration, preprocessing, and extraction of 3D photocephalometric measurements is made publicly available as a free and open-source tool (CraniumPy) on Github.^27^

## PATIENTS AND METHODS

### Patient Characteristics

A total of 408 patients born between 1991 and 2019 and diagnosed as having nonsyndromic sagittal synostosis, who underwent FBR, ESC, or SAC in our hospital and had at least one postoperative 3D photogrammetry image taken before age 6, were considered for this study. The 3D images were captured using a 3dMDhead setup. No hairstyling products are allowed on the day of imaging, and in the case of long hair, the hair needs to be loose and combed flat. Before acquisition, a special nylon cap is pulled tightly over the head to minimize hair-induced deformations. Images in which the head shape was camouflaged by hair were excluded during data collection.

Preoperative measurements were used to assess whether preoperative differences among the groups were present.

The study protocol was approved by the institution’s medical ethics committee (MEC-2016-312) and followed the statements of the Declaration of Helsinki.

### Treatment Protocol

The protocol in the Erasmus MC Sophia Children’s Hospital has changed over the past 15 years. Until 2002, all patients presenting with sagittal synostosis underwent FBR between the ages of 9 and 12 months regardless of their age at presentation. However, a relatively high incidence of preoperative papilledema (9%) was observed in patients who presented early and had to wait for surgery.^28^ Between 2002 and 2010, the ESC was introduced for children who presented before age 6 months. In 2010, we transitioned from ESC to SAC to reduce blood loss and extensiveness. Patients presenting after age 6 months undergo an FBR shortly after referral. More details about the three surgical techniques and clinical outcomes are presented in earlier studies.^10,12,29^

After surgery, patients have routine follow-up, involving skull radiographs, 3D photogrammetry, funduscopy, and OFC measurements at regular intervals.^30^

### 3D Photocephalometrics and Mean Cranial Shapes

The 3D images captured during at least one of six follow-up periods (FU1 through FU6) were included, as follows:

- FU1: 3 months postoperatively and age less than 18 months
- FU2: 24 months of age
- FU3: 36 months of age
- FU4: 48 months of age
- FU5: 60 months of age
- FU6: 72 months of age

**Fig. 2.**
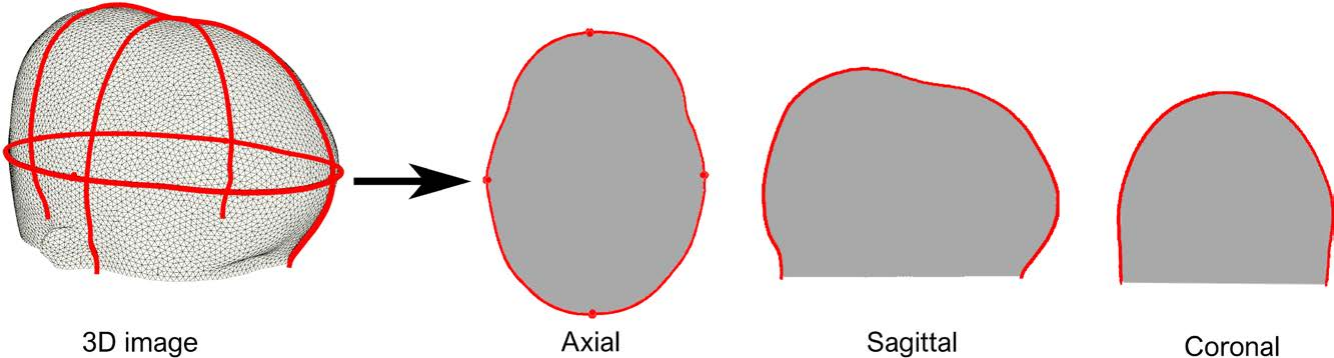
Extracted orthogonal slices from a three-dimensional image.

Measurements included the following:

- Maximum occipitofrontal diameter (OFD)
- Biparietal diameter
- OFC
- Orthogonal cranial slices (Fig. 2)
- Approximated ICV

A one-to-one translation from 3D photogrammetry to clinical measurements is reliable for measurements that are obtained in the same manner in clinic (OFC and cephalic index).^25^ However, volumetric measurements result in an overestimation of the intracranial volume and require a correction. This correction is based on reported correction factors in the literature and confirmed by strong linear correlation (R^2^ = 0.96) between ICV from computed tomography and 3D photogrammetry observed in a subset of patients who had a computed tomography scan acquired on the same day as their 3D photogrammetry image (*n* = 25).^9,31–33^

The reference plane in our pipeline is defined by the plane going through the nasion and both tragi. The centroid of these three landmarks serves as the initial anchor point and guides the registration process. [**See Figure, Supplemental Digital Content 1**, which shows (*left*) three selected landmarks (*nasion*, *left tragus*, *right tragus*) and their corresponding centroid. (*Center*) Transformation from source to template involves a translation of the center of mass and three rotations (*x*, *y*, *z*) around the orthogonal unit vectors. (*Right*) Center of mass translation is based on the extracted axial slice containing the largest head circumference, http://links.lww.com/PRS/G131.] To extract measurements, an iterative algorithm searched along slices parallel to the nasion–tragi plane. After locating the slice containing the largest OFD, an axial slice was extracted from the mesh (Fig. 2).

Measurements were converted to *z* scores before statistical testing. The *z* score describes how far each measurement is from its normocephalic, age- and sex-associated mean, expressed in SD. OFC measurements were converted to *z* scores using Growth Analyser with reference data by Talma et al.^34^ The *z* scores for ICV and cephalic index were calculated based on normal data presented by Abbott et al.^35^ and Waitzman et al.,^36^ respectively. Complementary to the statistical comparison of measurements, mean cranial shapes were generated along three orthogonal slices.

**Fig. 3.**
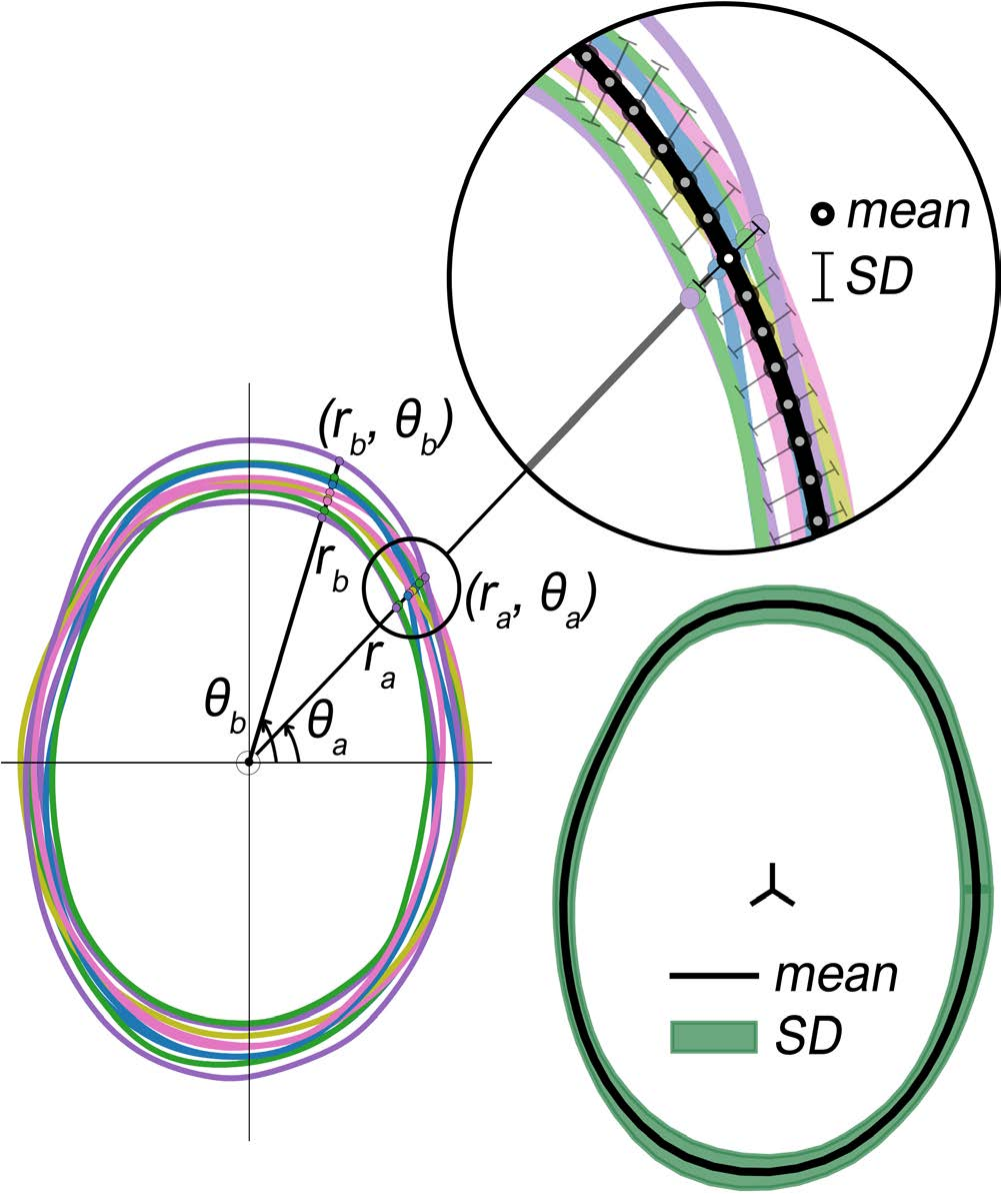
Overlaid axial slices extracted from different three-dimensional images (*left*). Generated mean shape and corresponding SDs (*right*).

Sagittal and coronal slices (Fig. 2) perpendicular to the axial OFD slice were extracted from every mesh. For 120 sampled points on every slice, a mean and SD was calculated (Fig. 3, *left*) and allowed us to generate mean cranial shapes (Fig. 3, *right*) for different techniques and age groups. A healthy age-related normal model was used as a reference.^37^ For further descriptions, **see Document, Supplemental Digital Content 2**, which shows the preprocessing steps of the 3D photogrammetry images, http://links.lww.com/PRS/G132.

### Statistical Analysis

Scipy Statistics was used for statistical analysis.^38^ Continuous variables were compared using the one-way analysis of variance test, after the assumptions of normality (Shapiro-Wilk test) and homogeneity of variance (Levene test) were confirmed. (**See Table, Supplemental Digital Content 3**, which shows statistical test selection for continuous variables, http://links.lww.com/PRS/G133.) The Kruskal-Wallis H test was used to compare continuous variables for which these assumptions were not true. A significance level less than 0.05 was considered significant.

## RESULTS

### Patient and Operative Characteristics

**Fig. 4.**
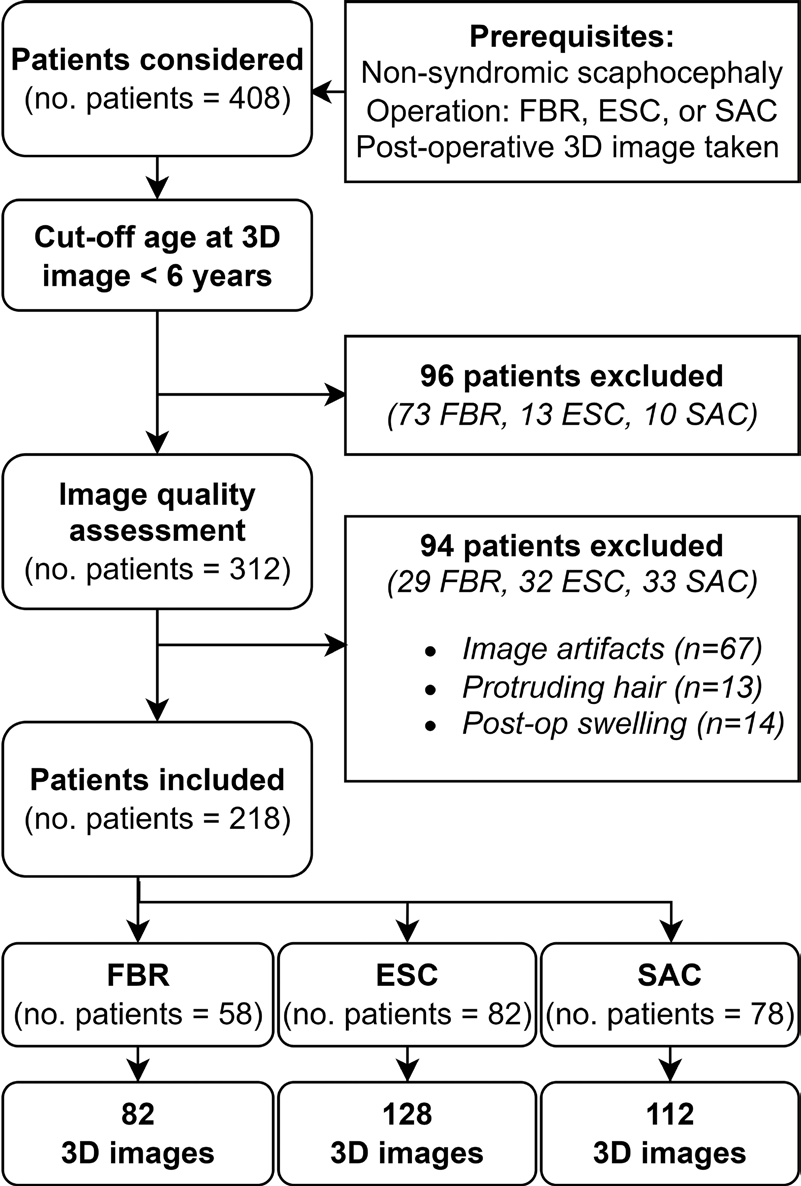
Flowchart of study inclusion criteria. *ESC*, extended strip craniectomy; *FBR*, frontobiparietal remodeling; *SAC*, spring-assisted correction.

After considering all prerequisites and exclusion criteria (Fig. 4), 218 patients (58 FBR, 82 ESC, 78 SAC) with a total of 322 3D images were included in this study (Table 1). In all three groups, there were more male than female patients, which is in line with the epidemiology of nonsyndromic sagittal synostosis.^2^

**Table 1.** Patient Characteristics^a^

Characteristics	FBR	ESC	SAC	Overall
No. of patients	58	82	78	218
Female	12 (20.7%)	10 (12.2%)	13 (16.7%)	35 (16.1%)
Male	46 (79.3%)	73 (87.8%)	65 (83.3%)	184 (83.9%)
3D images, *n*	82	128	112	322
Age at 3D image follow-up				
3 mo postop (FU1)	15.09 (13.75–15.82)	8.48 (7.99–9.18)	9.40 (8.65–10.09)	
3D images, *n*	18	48	13	
24 mo (FU2)	23.92 (23.19–24.43)	24.33 (20.73–27.35)	24.49 (23.86–25.40)	
3D images, *n*	16	26	26	
36 mo (FU3)	37.33 (37.17–39.08)	33.99 (31.28–36.31)	36.61 (35.88–37.50)	
3D images, *n*	8	11	34	
48 mo (FU4)	47.90 (47.47–49.84)	47.44 (46.42–49.12)	49.97 (48.00–50.20)	
3D images, *n*	20	21	17	
60 mo (FU5)	59.15 (58.96–60.01)	60.99 (59.53–62.18)	61.22 (60.56–61.94)	
3D images, *n*	3	4	9	
72 mo (FU6)	72.26 (71.84–73.97)	72.59 (71.15–75.08)	73.64 (72.20–75.45)	
3D images, *n*	17	18	13	

a Values are expressed as *n* (%) or median (interquartile range).

Operative characteristics and complications are presented in Table 2 and Table 3, respectively. The length of surgery was significantly different among the three surgical techniques. FBR surgery showed more extensive blood loss compared with ESC and SAC. Dural defects occurred in nine patients: seven in the FBR group and two in the SAC group.

**Table 2.** Operative Characteristics^a^

Characteristics	No. of Patients Evaluated	FBR (*n* = 58)	ESC (*n* = 82)	SAC (*n* = 78)	Overall (*n* = 218)	*P*
Age at surgery, mo	218	11.55 (10.51–12.64)	4.90 (4.31–5.5)	5.75 (5.41–6.00)	5.77 (5.1–9.13)	<0.001^b^
Surgery time, min	218	296.5 (269.25–329)	230 (205.5–258)	198.5 (174–222.5)	234 (198–275)	<0.001^b^
Blood loss, mL	207 (57 FBR,78 ESC,72 SAC)	600 (415–1000)	150 (100–300)	70 (43.8–121.3)	153.5 (80–400)	<0.001^b^

a Values are expressed as median (interquartile range).

b Kruskal-Wallis rank sum test (post hoc Conover test).

**Table 3.** Complication Frequency^a^

Complication	No. of Patients Evaluated	FBR (*n* = 58)	ESC (*n* = 82)	SAC (*n* = 78)	Overall
Disturbed wound healing	218	1 (1.7)	0 (0.0)	1 (1.3)	2 (0.9)
Dural tear	218	7 (12.1)	0 (0.0)	2 (2.6)	9 (4.1)
Infection	218	3 (5.2)	3 (3.7)	3 (3.8)	9 (4.1)
Hematoma	218	0 (0.0)	1 (1.2)	0 (0.0)	1 (0.5)

a Values are expressed as *n* (%).

### ICH and Reinterventions

Fifteen patients (five FBR, eight ESC, two SAC) had a reintervention because of ICH, skull defect, hematoma, or persistent scaphocephalic head shape. (**See Table, Supplemental Digital Content 4**, which shows ICH and reinterventions, shttp://links.lww.com/PRS/G134.) Patients who had a reintervention because of ICH underwent biparietal remodeling. Patients with skull defects underwent split-skull graft and patients with a persisting scaphocephalic shape underwent FBR.

In nine patients (two FBR, six ESC, one SAC), intracranial pressure was measured because of persistent papilledema. In six of those patients (one FBR, four ESC, one SAC), ICH was confirmed. A reintervention to reduce intracranial pressure was necessary in five out of the six patients. One patient did not have surgery because of disappearance of the papilledema; surgery was canceled, and watchful waiting was maintained.

Reinterventions because of skull defects were performed in four patients treated with FBR and three patients with ESC. A single patient treated with ESC required a reintervention because of a postoperative hematoma.

### 3D Photocephalometrics and Mean Cranial Shapes

Preoperative measurements from skull radiographs (cephalic index) and manual measurements (OFC) were used to determine a preoperative baseline. We observed no significant differences in preoperative cephalic index and OFC among the three groups after correcting for age and sex. (**See Table, Supplemental Digital Content 5**, which shows preoperative baseline evaluation, http://links.lww.com/PRS/G135.) Preoperative ICV measurements were not available. However, OFC has been shown to be a good proxy for ICV.^39^ This was verified using postoperative ICV and OFC measurements, which showed a strong correlation (R^2^ = 0.89). We therefore assumed a similar, not significant difference in preoperative ICV among the three groups.

**Fig. 5.**
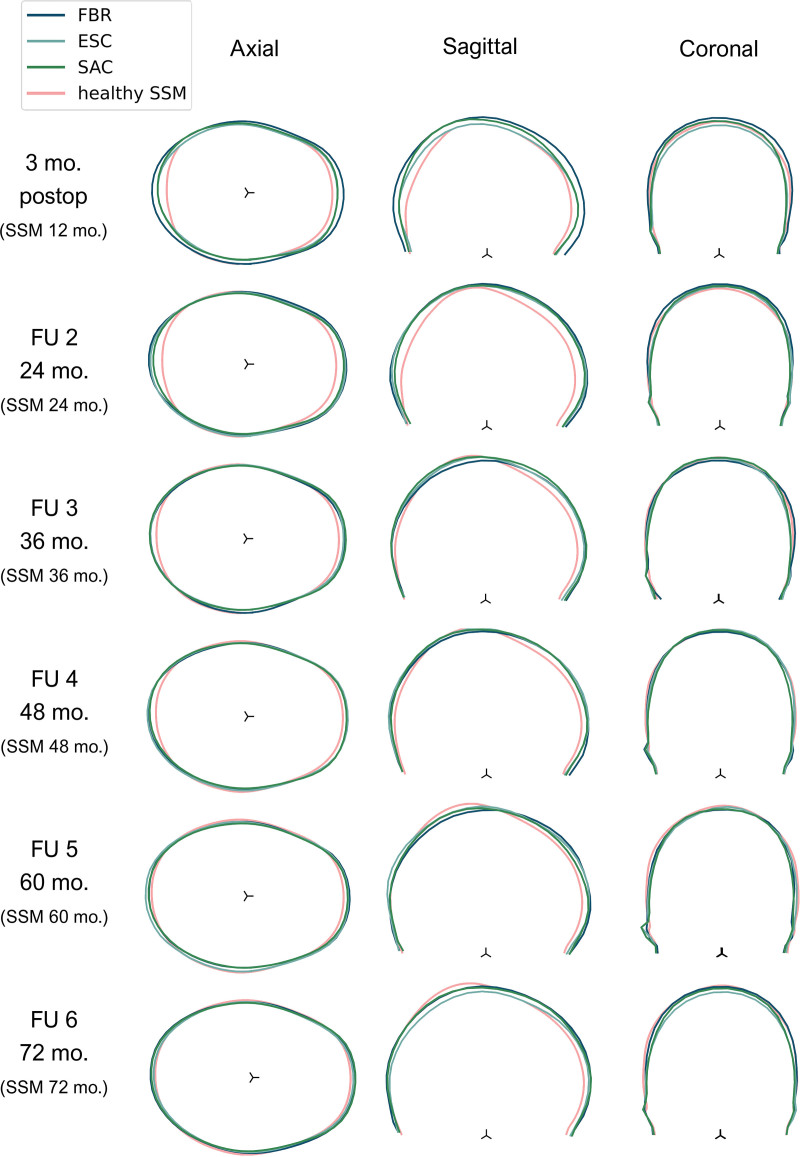
Mean postoperative cranial shapes from the six follow-up groups with reference to their age-specific normocephalic shape. *SSM*, statistical shape model.

**Fig. 6.**
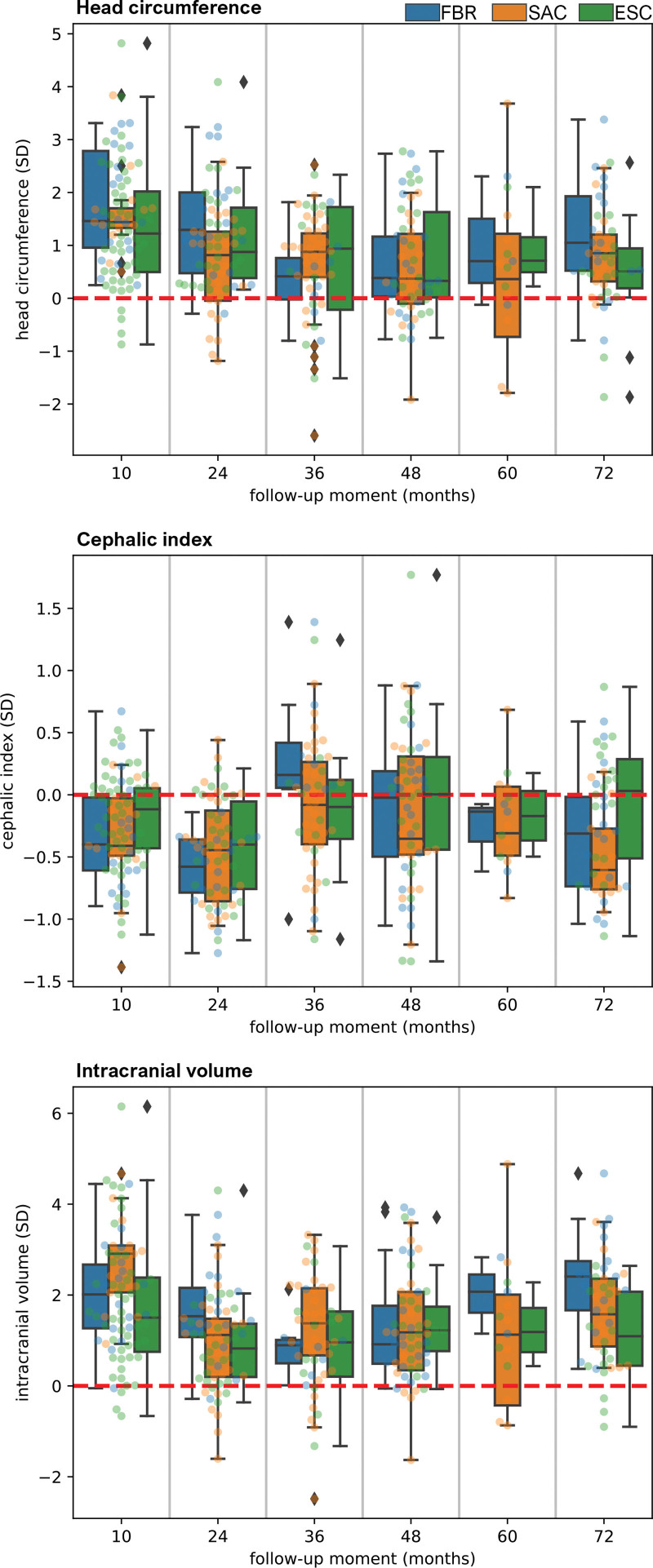
Photocephalometric measurements (*z* scores) from every operating group over time expressed in SDs. Head circumference (*above*). Cephalic index (*center*). Intracranial volume (*below*).

Mean postoperative cranial shapes with respect to normocephalic head shapes from a statistical shape model are presented in Figure 5.^37^ Extracted OFC, cephalic index, and ICV values are presented in Table 4 and plotted in Figure 6. Statistical testing showed no significant differences in *z* scores among the three groups, with the exception of ICV in the follow-up group at 72 months (Table 4). However, post hoc tests with a Bonferroni correction to correct for multiple comparisons did not show significant pairwise differences in ICV.

**Table 4.** Postoperative Photocephalometric Measurements^a^

Surgery Follow-Up Group	OFC		Cephalic Index		ICV	
	cm	*z* Score	%	*z* Score	cc	*z* Score
FU1: 3 mo postoperatively (3D images: *n* = 79)						
Total (79)	48.29 ± 1.83	1.49 ± 1.09; 1.42 (0.67–2.19)	75.32 ± 3.57	−0.24 ± 0.41; −0.22 (−0.5 to 0.05)	1204 ± 138	1.97 ± 1.35; 1.80 (0.99–2.88)
FBR (18)	50.26 ± 1.50	1.77 ± 1.05; 1.46 (0.96–2.78)	74.47 ± 3.87	−0.31 ± 0.44; −0.40 (−0.61 to −0.02)	1360 ± 94	2.01 ± 1.10; 2.01 (1.27–2.67)
ESC (48)	47.58 ± 1.56	1.35 ± 1.16; 1.22 (0.50–2.02)	75.53 ± 3.47	−0.19 ± 0.38; −0.12 (−0.43 to 0.05)	1138 ± 113	1.76 ± 1.45; 1.50 (0.75–2.38)
SAC (13)	48.18 ± 1.17	1.63 ± 0.83; 1.44 (1.33–1.70)	75.75 ± 3.59	−0.35 ± 0.45; −0.41 (−0.49 to −0.03)	1235 ± 84	2.70 ± 1.08; 2.91 (2.06–3.09)
* P*		0.289^b^		0.345^c^		0.080^c^
FU2: age 24 mo (3D images: *n* = 68)						
Total (68)	50.72 ± 1.67	0.99 ± 1.03; 1.03 (0.25 to 1.58)	73.50 ± 3.60	−0.48 ± 0.39; −0.45 (−0.79 to −0.25)	1408 ± 122;	1.08 ± 1.09; 1.13 (0.25–1.69)
FBR (16)	51.32 ± 1.92	1.43 ± 1.08; 1.29 (0.47 to 2.0)	72.35 ± 2.91	−0.62 ± 0.31; −0.58 (−0.79 to −0.36)	1463 ± 140)	1.62 ± 1.07; 1.53 (1.08–2.16)
ESC (26)	50.97 ± 1.44	1.13 ± 0.94; 0.87 (0.38 to 1.71)	73.89 ± 3.55	−0.42 ± 0.38; −0.40 (−0.76 to −0.05)	1394 ± 104)	0.96 ± 0.98; 0.82 (0.20–1.37)
SAC (26)	50.11 ± 1.59	0.59 ± 0.96; 0.82 (−0.05 to 1.26)	73.81 ± 4.00	−0.45 ± 0.42; −0.45 (−0.86 to −0.13)	1388 ± 122)	0.86 ± 1.13; 1.12 (0.20–1.48)
* P*		0.055^b^		0.239^c^		0.066^b^
FU3: age 36 mo (3D images: *n* = 53)						
Total (53)	51.32 ± 1.78	0.58 ± 1.01; 0.8 (0.06 to 1.27)	74.38 ± 3.78	−0.03 ± 0.52; 0.01 (−0.32 to 0.29)	1475 ± 138	1.05 ± 1.16; 1.0 (0.33–1.64)
FBR (8)	51.05 ± 1.51	0.41 ± 0.80; 0.41 (−0.02 to 0.76)	76.24 ± 5.03	0.23 ± 0.68; 0.16 (0.06 to 0.42)	1442 ± 95	0.85 ± 0.63; 0.89 (0.50–1.01)
ESC (11)	51.05 ± 1.74	0.55 ± 1.18; 0.50 (−0.32 to 1.67)	75.08 ± 3.66	−0.01 ± 0.51; 0.01 (−0.31 to 0.14)	1435 ± 120	0.74 ± 1.16; 0.58 (0.12–1.52)
SAC (34)	51.48 ± 1.87	0.63 ± 1.02; 0.88 (0.40 to 1.23)	73.72 ± 3.41	−0.11 ± 0.48; −0.08 (−0.40 to 0.26)	1495 ± 151	1.19 ± 1.25; 1.38 (0.67–2.15)
* P*		0.678^b^		0.252^c^		0.478^c^
FU4: age 48 mo (3D images: *n* = 58)						
Total (58)	52.25 ± 1.73	0.69 ± 1.02; 0.38 (−0.01 to 1.42)	73.82 ± 4.44	−0.12 ± 0.61; −0.06 (−0.48 to 0.25)	1544 ± 136	1.30 ± 1.17; 1.16 (0.51–2.0)
FBR (20)	52.24 ± 1.70	0.67 ± 1.01; 0.38 (0.03 to 1.17)	73.58 ± 3.80	−0.14 ± 0.53; −0.02 (−0.50 to 0.19)	1544 ± 136	1.29 ± 1.22; 0.92 (0.49–1.76)
ESC (21)	52.50 ± 1.74	0.85 ± 1.07; 0.39 (0.03 to 1.68)	74.04 ± 5.42	−0.09 ± 0.75; −0.01 (−0.55 to 0.28)	1552 ± 107	1.38 ± 0.94; 1.23 (0.83–1.87)
SAC (17)	51.96 ± 1.83	0.51 ± 1.02; 0.37 (−0.15 to 1.25)	73.83 ± 4.02	−0.12 ± 0.54; −0.31 (−0.47 to 0.37)	1536 ± 172	1.23 ± 1.39; 1.18 (0.29–2.08)
* P*		0.596^c^		0.963^c^		0.771^b^
FU5: age 60 mo (3D images: *n* = 16)						
Total (16)	52.33 ± 2.21	0.64 ± 1.39; 0.62 (0.03 to 1.22)	73.03 ± 3.67	−0.27 ± 0.44; −0.29 (−0.51 to 0.0)	1551 ± 149	1.37 ± 1.46; 1.30 (0.71 to 2.12)
FBR (3)	52.43 ± 1.63	0.96 ± 1.23; 0.70 (0.29 to 1.50)	72.8 ± 2.44	−0.28 ± 0.30; −0.14 (−0.38 to −0.11)	1558 ± 42	2.02 ± 0.84; 2.07 (1.61 to 2.45)
ESC (4)	53.10 ± 1.34	0.94 ± 0.82; 0.71 (0.49 to 1.15)	73.9 ± 2.48	−0.17 ± 0.30; −0.17 (−0.37 to 0.03)	1578 ± 93	1.27 ± 0.81; 1.19 (0.74 to 1.72)
SAC (9)	51.97 ± 2.71	0.40 ± 1.68; 0.37 (−0.42 to 1.10)	72.71 ± 4.57	−0.32 ± 0.55; −0.37 (−0.54 to 0.06)	1536 ± 193	1.20 ± 1.83; 1.15 (−0.31 to 1.77)
* P*		0.759^c^		0.871^c^		0.723^c^
FU6: age 72 mo (3D images: *n* = 48)						
Total (48)	53.09 ± 1.68	0.85 ± 0.98; 0.74 (0.38–1.34)	74.11 ± 3.53	−0.27 ± 0.49; −0.29 (−0.73 to 0.12)	1618 ± 141	1.68 ± 1.17; 1.78 (0.71–2.41)
FBR (17)	53.54 ± 1.90	1.15 ± 1.07; 1.05 (0.52–1.93)	73.78 ± 3.48	−0.32 ± 0.49; −0.31 (−0.74 to −0.02)	1684 ± 153	2.30 ± 1.12; 2.41 (1.66–2.75)
ESC (18)	52.52 ± 1.61	0.54 ± 0.98; 0.51 (0.19–0.94)	75.45 ± 3.82	−0.08 ± 0.52; 0.03 (−0.51 to 0.29)	1542 ± 109	1.09 ± 1.07; 1.09 (0.44–2.07)
SAC (13)	53.30 ± 1.34	0.90 ± 0.78; 0.85 (0.32–1.20)	72.69 ± 2.66	−0.46 ± 0.38; −0.60 (−0.76 to −0.27)	1638 ± 123	1.67 ± 1.03; 1.58 (0.87–2.36)
* P*		0.190^c^		0.085^c^		<0.05^c^; post hoc not significant

a Values are expressed as mean (SD); median (interquartile range).

b Kruskal-Wallis rank sum test (post hoc Conover test, Bonferroni correction).

c One-way analysis of variance (post hoc pairwise *t* test).

Early on, the cranial shapes in the axial and sagittal plane (Fig. 5) show that scaphocephalic features such as frontal bossing and occipital bullet persist until age 24 months, regardless of the operating technique. The data show that at 24 months, the mean OFC and ICV are 1 SD above normal, with a cephalic index of −0.5 SD. Over time, the cephalic index normalizes, as shown in Figure 5, with a mean cephalic index value of −0.03 SD at 36 months, −0.12 SD at 48 months, and −0.27 SD at 60 and 72 months. At 36, 48, 60, and 72 months, respectively, increased mean OFC values (+0.58 SD, +0.69 SD, +0.64 SD, +0.85 SD) and ICV values (+1.05 SD, +1.30 SD, +1.37 SD, +1.68 SD) are observed compared with normal.

Flattening of the vertex can be observed in the sagittal and coronal planes in FU5 and FU6 (Fig. 5), causing an anterior displacement of the position of maximum vertex height. The ESC FU6 group shows the lowest vertex with respect to the other two groups. The width of the skull is not evidently different from the normal population.

## DISCUSSION

This is one of the largest studies evaluating the three surgical techniques (ESC, FBR, and SAC) until age 6 based on both 3D photogrammetry and operative characteristics.

### Postoperative Outcomes

Many studies have compared surgical outcomes to determine the differences in surgical techniques based on cephalic index, OFC, and ICV. Bonfield et al.^18^ reported that cranial vault remodeling (CVR) and endoscopic-assisted craniectomy led to the largest improvement in cephalic index compared with other surgical techniques, including SAC and ESC. This larger effect is possibly explained by a lower preoperative cephalic index in the CVR and endoscopic-assisted craniectomy groups, according to the authors.^18^ Differences in OFC between techniques vary within the literature. De Praeter et al.^19^ showed a larger increase in OFC for CVR compared with ESC in a small study. However, we have not found significant differences in postoperative cephalic index and OFC among the techniques (Table 4), which is in line with the majority of comparable studies.^17,20,40–42^

Fischer et al.^43^ and Mertens et al.^44^ indicated no differences in ICV measures after SAC or ESC compared with pi-plasty surgery. Arab et al.^45^ concluded that extensive cranioplasties resulted in a smaller ICV, whereas SAC and ESC combined did not show these results. The problem with a smaller ICV is that it might be related to the development of ICH, an important complication seen in patients with craniosynostosis.^46–48^ Our results showed no differences in *z* scores of postoperative ICV in the first five follow-up groups. Relatively large differences in ICV were observed in the final follow-up group at age 72 months (Table 4). Pairwise post hoc tests were unable to detect significant differences, which may be caused by a low statistical power. These differences may be clinically relevant with regard to the long-term effects of the surgical techniques. For example, could the smaller ICVs in the ESC group relate to an increase in hypertension, or the relatively large ICV in the FBR group result in other complications, later in life? To obtain conclusive answers to these questions, larger studies and collaborations are required.

When we look at the postoperative outcomes in comparison with the normative population, we see a clear normalization of the cephalic index in all three groups, but both OFC and ICV values were consistently higher than normal for age. We hypothesize that this is because the three techniques focus on harmonization of craniofacial proportions, attaining a near normal cephalic index by widening rather than shortening of the head when correcting the scaphocephalic shape. With an above-average head depth inherent to this condition, this “harmonization” inevitably leads to an increased OFC and ICV value compared with normal. The persistence of larger than normal OFC and ICV values may suggest that normative growth potential is not impaired by these interventions. Sgouros et al.^49^ reported similar results in a study on postoperative ICV development in craniosynostosis and observed that these children followed a growth curve parallel to that of healthy children with a considerably higher volume. A significantly larger than normal OFC and ICV were also reported by Toma et al.^50^ after total vault remodeling.

The generated mean cranial shapes (Fig. 5) show that all three techniques generally correct the distinctive scaphocephalic features, such as frontal bossing and occipital bulging. The observable differences in mean shape among the three operating groups in the first two follow-up periods could be explained by the significant difference in mean age at surgery (Table 2) instead of an inherent effect of a particular operating technique. Longitudinal visualizations of mean shapes for every operating technique confirm this discrepancy between the first and second follow-up group. (**See Figure, Supplemental Digital Content 6**, which shows mean postoperative cranial shape development over time for every operating group, http://links.lww.com/PRS/G136.) Frontal and occipital regions correct over time, irrespective of the inclusion of the forehead or occiput in the remodeling. Flattening of the vertex seems to persist after surgical correction (Fig. 5; FU5 and FU6). Correcting the position of the vertex remains a challenge and may guide future modifications of surgical techniques.

The mean shape visualizations corroborate our statistical results that postoperative differences among operating techniques are limited and show that more comprehensive measures are required to evaluate the cranial morphology and all its intricacies in three dimensions.

### Importance of Early Diagnosis and the Potential of 3D Photogrammetry

Our findings show that FBR is associated with a longer mean surgery time, an increased risk of dural defects, and higher blood loss compared with ESC and SAC (Table 2). This result is in line with other reports and favors early minimal intervention above late extensive surgery. Because the age at presentation is the decisive factor for the type of surgery a patient receives, it is important to emphasize the importance of an early diagnosis. In addition to increasing awareness about the early signs of craniosynostosis, the development of novel diagnostic tools may be helpful early on. When craniosynostosis is suspected, a patient always has to be referred to a craniofacial center for further examination and diagnosis.

Novel machine-learning methods for classifying and quantifying different types and severities of craniosynostoses based on 3D photogrammetry data have shown good results.^51,52^ Next steps may involve the use of deep-learning methods, such as autoencoders based on mesh convolution operators.^53,54^

### Study Limitations

Data were not distributed evenly within the FU groups. It is important, therefore, to consider the number of samples used to generate the mean shapes, as well as differences between age groups, when interpreting the results.

We demonstrated that 3D photogrammetry can be used for rapid automatic extraction of measurements, without the need for labor-intensive measurements and invasive imaging modalities. However, the complexity of cranial development makes finding a stationary reference point for craniofacial analysis challenging, particularly when landmarks are limited to distinct features on the surface. Our reference point, based on the center of mass, is easy to reproduce and provides relevant information about the skull shape development. This reference point will likely be less suitable for the detection of anisotropic growth effects (eg, excessive anterior growth), because these effects will be averaged out when using the center of mass.

## CONCLUSIONS

No statistically significant differences in cephalic index, OFC, or ICV were observed among the surgical interventions. FBR has a longer mean surgery time and shows a larger number of dural defects and higher blood loss than ESC and SAC. Because age at presentation is the main determinant on the basis of which minimally invasive surgery can be considered, early diagnosis is important. Three-dimensional photogrammetry offers the opportunity to acquire high-dimensional, longitudinal data for retrospective analysis, and can be a promising way forward in the early detection of craniofacial dysmorphologies and to enhance personalized treatment. As a part of this study, our 3D image-processing tool has been made publicly available for preprocessing of 3D meshes and extraction of 3D photocephalometric measurements in a quick, accessible, and reproducible manner.

## DISCLOSURE

The authors have no competing interests or any financial interest to declare in relation to the content of this article.

## ACKNOWLEDGMENT

The authors would like to thank Nicole Erler, biostatistician in the Erasmus MC, who was readily available for questions and discussions regarding the statistical analysis of this study.

## Supplementary Material
