# Supplementary figures and images for: Sagittal Craniosynostosis: Comparing Surgical Techniques Using 3D Photogrammetry
Source: Plast Reconstr Surg. 2023 Mar 22;152(4):675–88. doi: 10.1097/PRS.0000000000010441 (PMC10521803; doi:10.1097/PRS.0000000000010441)

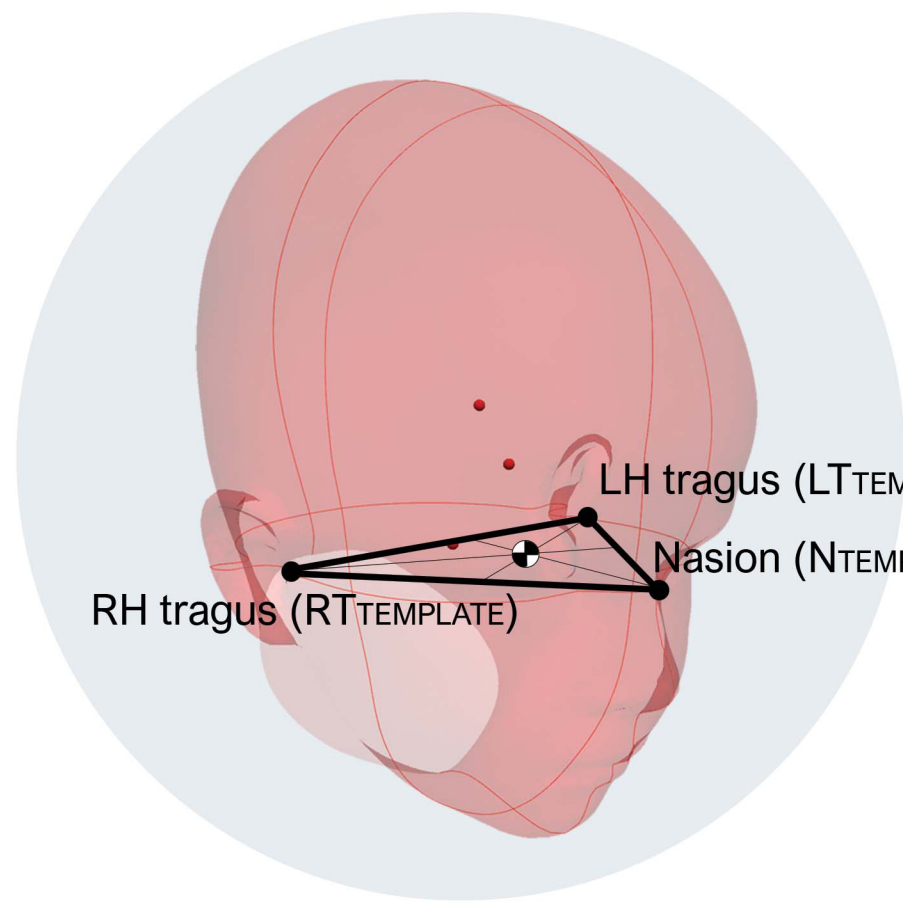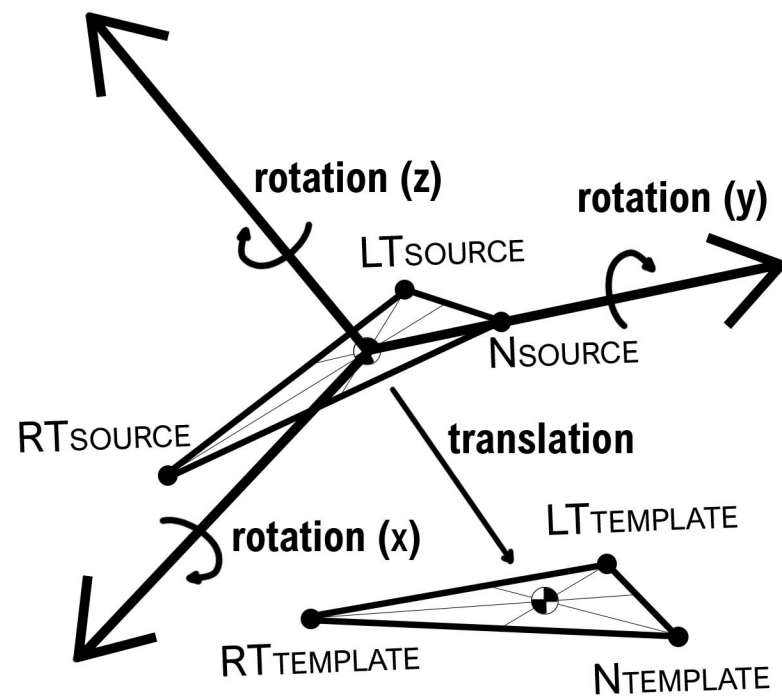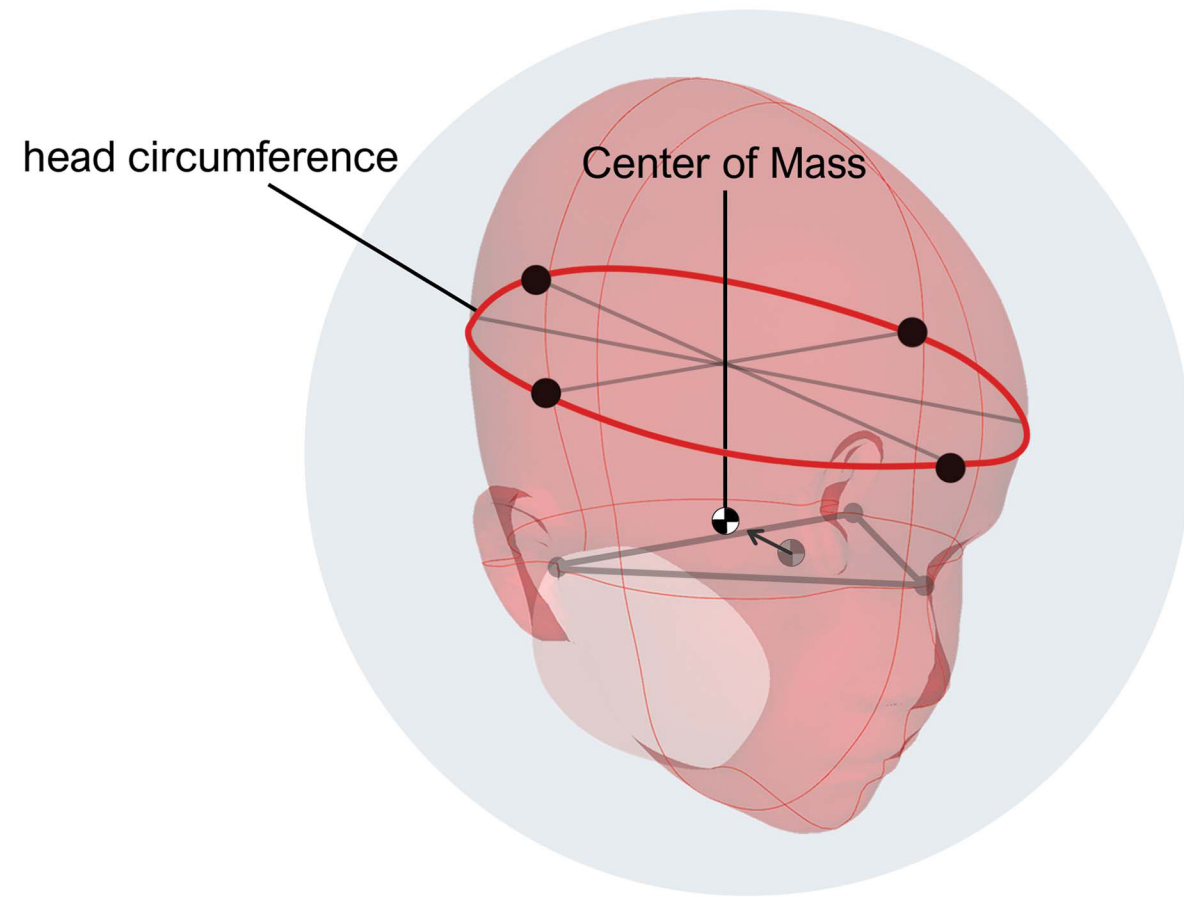

Supplement: Supplementary file 1 [file prs-152-675e-s001.pdf]

Axial

Sagittal

Coronal

FBR

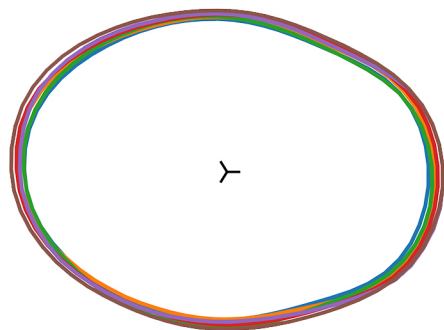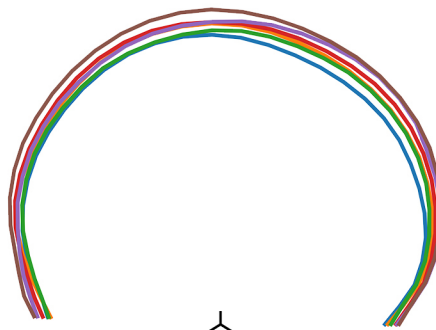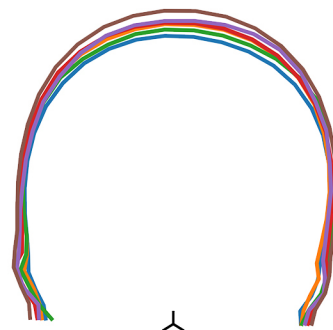

ESC

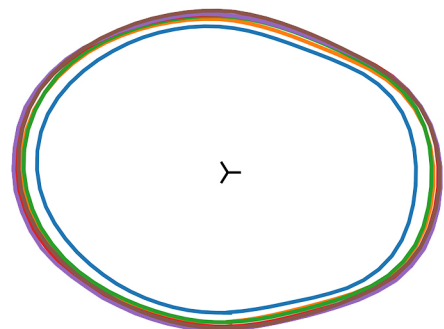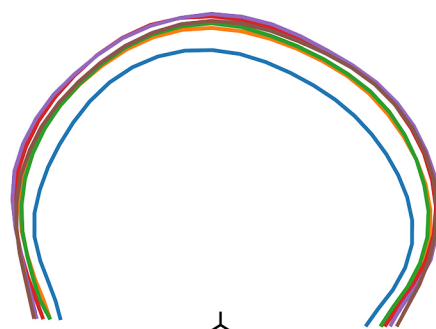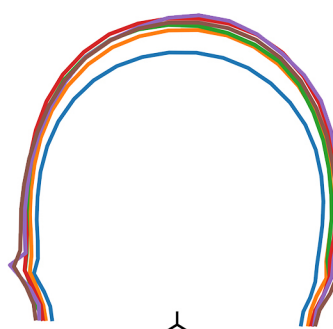

SAC

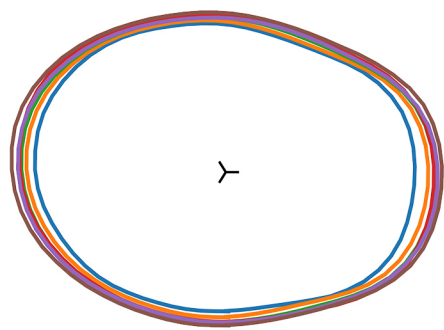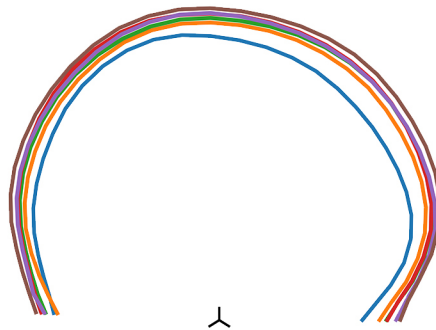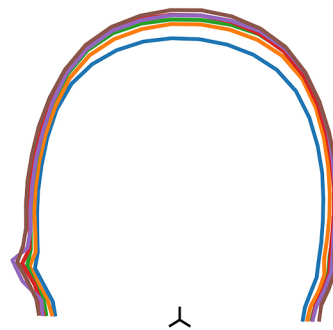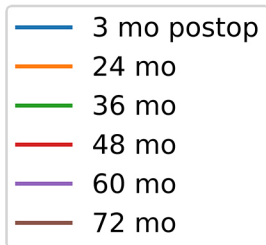

Supplement: Supplementary file 6 [file prs-152-675e-s006.pdf]
