# Supplementary material for: Sagittal Craniosynostosis: Comparing Surgical Techniques Using 3D Photogrammetry
Source: Plast Reconstr Surg. 2023 Mar 22;152(4):675–88. doi: 10.1097/PRS.0000000000010441 (PMC10521803; doi:10.1097/PRS.0000000000010441)
Supplement: Supplementary file 2 [file prs-152-675e-s002.pdf]

Supplemental Digital Content 2

Pre-processing of 3D photogrammetry images

The nasion and both tragi are manually identified, after which meshes are automatically registered to a healthy template from a statistical shape model.[1] For this registration step and further analysis, a standardized reference frame for aligning 3D images is essential. The origin of this reference frame should be defined such that it represents a stable point in the cranium that does not shift during growth. Several reference planes for 3D photogrammetry based on external landmarks have been used in literature.[2–6]

For our study, we selected the nasion-tragi plane as our frame of reference. The centroid of the three landmarks (nasion, left tragus, and right tragus) serves as the initial anchor point and guide the image registration process.

The required translation of the mesh is described by the Euclidian distance between the landmark centroid of the source mesh (i.e. mean $[x, y, z]$ position of the three selected landmarks) and the landmark centroid of the target (template) mesh. Iteratively, the mesh is rotated along each axis until the three vectors are aligned with the corresponding vectors on the template. Because the centroid of the three landmarks is not a direct function of the posterior region, the center of mass was extracted from the head circumference slice. The discrepancy between the initial centroid and the center of mass was calculated, and finally a translation was applied to the mesh (in anterior-posterior direction). This translated centroid is located at the origin of the frame of reference $([x, y, z] = [0,0,0])$.

The shape and number of mesh elements influence the accuracy of the 3D representation. For comparative analysis, resampling of the meshes is performed to model each 3D image with the same number of uniformly distributed triangular elements using Voronoi clustering implemented in the *pyacvd* library.[7] Mesh interaction was mainly based on the PyVista library.[8] Small mesh defects are automatically repaired using an iterative repair algorithm that can detect and remove undesired elements, returning a water tight model.[9]

1. T. Huysmans, L. Goto, J. Molenbroek RG. DINED Mannequin. *Tijdschr voor Hum Factors*. 2020;45(1):4--7.
2. McKay DR, Davidge KM, Williams SK, et al. Measuring cranial vault volume with three-dimensional photography: A method of measurement comparable to the gold standard. *J Craniofac Surg*. 2010;21(5):1419-1422. doi:10.1097/SCS.0b013e3181ebe92a
3. Seeberger R, Hoffmann J, Freudlsperger C, et al. Intracranial volume (ICV) in isolated sagittal craniosynostosis measured by 3D photocephalometry: A new perspective on a controversial issue. *J Cranio-Maxillofacial Surg*. 2016;44(5):626-631. doi:10.1016/j.jcms.2016.01.023
4. Martini M, Klausing A, Lüchters G, Heim N, Messing-Jünger M. Head circumference - a useful single parameter for skull volume development in cranial growth analysis? *Head Face Med*. 2018;14(1):3. doi:10.1186/s13005-017-0159-8
5. Weinberg SM, Naidoo S, Govier DP, Martin RA, Kane AA, Marazita ML. Anthropometric precision and accuracy of digital three-dimensional photogrammetry: Comparing the genex and 3dMD imaging systems with one another and with direct anthropometry. *J Craniofac Surg*. 2006;17(3):477-483. doi:10.1097/00001665-200605000-00015
6. Schaaf H, Pons-Kuehnemann J, Malik CY, et al. Accuracy of three-dimensional photogrammetric images in non-synostotic cranial deformities. *Neuropediatrics*. 2010;41(1):24-29. doi:10.1055/s-0030-1255060
7. pyacvd. https://github.com/pyvista/pyacvd
8. Sullivan CB, Kaszynski A. PyVista: 3D plotting and mesh analysis through a streamlined interface for the Visualization Toolkit (VTK). *J Open Source Softw*. 2019;4(37):1450. doi:10.21105/joss.01450
9. Attene M. A lightweight approach to repairing digitized polygon meshes. *Vis Comput*. 2010;26(11):1393-1406. doi:10.1007/s00371-010-0416-3
