# Supplementary material for: Sagittal Craniosynostosis: Comparing Surgical Techniques Using 3D Photogrammetry
Source: Plast Reconstr Surg. 2023 Mar 22;152(4):675–88. doi: 10.1097/PRS.0000000000010441 (PMC10521803; doi:10.1097/PRS.0000000000010441)
Supplement: Supplementary file 3 [file prs-152-675e-s003.pdf]

Follow-up group<br>No. of samples	Shapiro-Wilk test<br>P > 0.05:<br>Gaussian distribution<br>|FBR|ESC|SAC|	Levene's test<br>P > 0.05:<br>Equal variance	One-way Anova	Kruskal-Wallis test
**FU1**	**OFC** |0.128|0.489|0.033|	0.180		X
**3 months postop**	**CI** |0.322|0.415|0.275|	0.752	X	
**FBR: 18, ESC: 48, SAC: 13**	**ICV** |0.984|0.053|0.974|	0.495	X	
**FU2**	**OFC** |0.440|0.033|0.419|	0.665		X
**24 months**	**CI** |0.456|0.378|0.188|	0.501	X	
**FBR: 16, ESC: 26, SAC: 26**	**ICV** |0.985|0.006|0.821|	0.677		X
**FU3**	**OFC** |0.994|0.137|0.013|	0.418		X
**36 months**	**CI** |0.430|0.153|0.991|	0.859	X	
**FBR: 8, ESC: 11, SAC: 34**	**ICV** |0.386|0.950|0.348|	0.217	X	
**FU4**	**OFC** |0.144|0.059|0.620|	0.815	X	
**48 months**	**CI** |0.711|0.570|0.125|	0.564	X	
**FBR: 20, ESC: 21, SAC: 17**	**ICV** |0.006|0.279|0.956|	0.286		X
**FU5**	**OFC** |0.651|0.375|0.681|	0.506	X	
**60 months**	**CI** |0.196|0.803|0.980|	0.475	X	
**FBR: 3, ESC: 4, SAC: 9**	**ICV** |0.892|0.853|0.454|	0.345	X	
**FU6**	**OFC** |0.973|0.225|0.475|	0.552	X	

**72 months**	**CI** |0.710|0.639|0.105|	0.603	X	
**FBR: 17, ESC: 18, SAC: 13**	**ICV** |0.958|0.489|0.570|	0.952	X	

Table, SDC 3. Statistical test selection for continuous variables
