# Supplementary material for: Sagittal Craniosynostosis: Comparing Surgical Techniques Using 3D Photogrammetry
Source: Plast Reconstr Surg. 2023 Mar 22;152(4):675–88. doi: 10.1097/PRS.0000000000010441 (PMC10521803; doi:10.1097/PRS.0000000000010441)
Supplement: Supplementary file 4 [file prs-152-675e-s004.pdf]

	FBR (n=58)	ESC (n=82)	SAC (n=78)	Overall (n=218)
**ICH**	1 (2.0%)	4 (5.4%)	1 (1.4%)	6 (3.1%)
**Re-intervention**	5 (8.6%)	8 (9.6%)	2 (2.6%)	15 (6.8%)
**ICH**	1	3	1	5
**Skull defect**	4	3	-	7
**Persisting scaphocephalic shape**	-	1	1	2
**Hematoma**	-	1	-	1

Table, SDC 4. ICH and re-interventions
