# Supplementary material for: Sagittal Craniosynostosis: Comparing Surgical Techniques Using 3D Photogrammetry
Source: Plast Reconstr Surg. 2023 Mar 22;152(4):675–88. doi: 10.1097/PRS.0000000000010441 (PMC10521803; doi:10.1097/PRS.0000000000010441)
Supplement: Supplementary file 5 [file prs-152-675e-s005.pdf]

	FBR	ESC	SAC	Overall	p-value
**No. of measurements** [1]	31	61	57	149	
**OFC (cm)**					
Mean (SD)	47.43 (2.17)	42.81 (2.13)	43.04 (1.93)	43.86 (2.75)	
**OFC (z-score)**					
Mean (SD)	2.10 (1.77)	1.65 (1.32)	1.92 (0.94)	1.85 (1.31)	
Median	2.14	1.95	1.95	1.95	0.501 [3]
[IQR]	[0.92-2.87]	[0.88-2.38]	[1.18-2.52]	[1.06-2.52]	
**No. of measurements** [2]	12	32	15	59	
**CI (%)**					
Mean (SD)	66.52 (3.93)	66.17 (3.59)	67.45 (3.01)	66.57 (3.51)	
**CI (z-score)**					
Mean (SD)	-1.49 (0.55)	-1.38 (0.53)	-1.25 (0.32)	-1.36 (0.49)	0.469 [4]
Median	-1.45	-1.50	-1.23	-1.36	
[IQR]	[-1.86- -1.12]	[-1.78- -0.90]	[-1.45- -1.14]	[-1.76- -1.07]	

1. **Measurements obtained in clinic (measuring tape)**
2. **Measurements obtained from preoperative skull radiographs**
3. **Kruskal-Wallis rank sum test**
4. **One-way ANOVA**

Table, SDC 5. Preoperative baseline evaluation
